# Effect of 3D-printed model base height on thickness distribution of thermoformed zendura FLX dual-layer clear aligners: an *in vitro* study

**DOI:** 10.3389/fdmed.2026.1889969

**Published:** 2026-09-18

**Authors:** Thi-Bich-Van Tran, Ngoc-Anh-Thu Pham

**Affiliations:** Faculty of Dentistry, University of Medicine and Pharmacy at Ho Chi Minh City, Vietnam

**Keywords:** clear aligners, dual-layer aligners, model base height, thermoforming, thickness distribution

## Abstract

**Background:**

Thermoforming of clear aligners causes non-uniform material thinning that may influence the structural and mechanical characteristics of the aligners. While greater model base height (MBH) has been shown to increase thinning in single-layer aligners, its effect on dual-layer materials remains unclear. This study aimed to evaluate the effect of four MBH levels (2, 4, 6, and 8 mm) on the thickness distribution of thermoformed Zendura FLX dual-layer clear aligners.

**Methods:**

Forty Zendura FLX aligners were thermoformed over standardized 3D-printed maxillary models with MBH of 2, 4, 6, and 8 mm (*n* = 10 per group). Thickness was measured at multiple tooth-specific sites across four representative teeth (central incisor, canine, first premolar, and first molar) using a digital caliper. Linear mixed-effects models and generalized linear mixed-effects models with a gamma distribution were used for statistical analysis. Estimated marginal means (EMMs) and 95% confidence intervals (CIs) were derived from the fitted models to summarize group-level thickness estimates. *Post-hoc* pairwise comparisons were performed using Tukey's test, with significance set at *p* < 0.05.

**Results:**

EMM thickness decreased progressively with increasing MBH: 446 µm (95% CI 432–460) at MBH 2 mm, 386 µm (95% CI 374–399) at MBH 4 mm, 329 µm (95% CI 319–340) at MBH 6 mm, and 268 µm (95% CI 260–277) at MBH 8 mm (all pairwise comparisons *p* < 0.001). This decreasing trend was consistent across all tooth types and measurement sites, though the magnitude of reduction varied by site. Palatal sites consistently exhibited greater thickness than facial sites. For most tooth type–site combinations, all MBH contrasts were statistically significant, with limited exceptions observed at specific sites for anterior teeth, molars, and premolars.

**Conclusions:**

Increasing MBH resulted in a progressive reduction in the thickness of thermoformed Zendura FLX dual-layer clear aligners across all tooth types and measurement sites. Among the four levels tested, an MBH of approximately 4 mm appeared to provide a more balanced thickness distribution. These findings suggest that MBH should be considered as an important fabrication parameter in clear aligner production.

## Background

Clear aligner therapy (CAT) has become increasingly popular in orthodontic practice due to its perceived advantages, including favorable aesthetics, improved patient comfort, and removability compared with conventional fixed appliances (1). However, clinical outcomes remain variable. Recent studies have shown that the biomechanical forces and moments delivered by aligners often differ from those predicted in virtual treatment planning, indicating limited biomechanical predictability of current aligner systems (2, 3).

A principal contributor to this variability is the non-uniform thickness distribution of thermoformed aligners. During fabrication, uneven stretching of the heated thermoplastic sheet leads to localized thinning, which may alter the mechanical behavior and structural characteristics of the aligners (4, 5). Previous studies have mainly focused on single-layer polyethylene terephthalate glycol (PETG) aligners, consistently reporting significant thinning in curved and cervical regions (6, 7). In contrast, evidence on dual-layer or hybrid aligner materials, which possess different mechanical properties and may respond differently to thermoforming, remains limited.

Dual-layer aligners may respond differently to thermoforming than conventional single-layer materials because they consist of two polymers with different mechanical properties (8). The PETG outer layer provides rigidity and dimensional stability, whereas the TPU inner layer provides greater elasticity and resilience. During thermoforming, these differences may influence how the material stretches over the dental model, resulting in thickness distributions that differ from those of single-layer PETG aligners (9–12).

Dual-layer aligner materials such as Zendura FLX, which comprises a rigid PETG outer layer bonded to a flexible thermoplastic polyurethane (TPU) inner layer, have been developed with the aim of improving force sustainability and wear resistance during prolonged clinical use (13). Aligner thickness influences its stiffness and may therefore affect the orthodontic forces delivered to teeth (11, 14). Although previous studies have shown that thicker aligners generally produce greater forces, no clear threshold has been established to determine how much thickness reduction becomes clinically meaningful (15–17). However, the influence of fabrication parameters, particularly model base height (MBH), on thickness distribution in dual-layer aligners has received limited attention. Although greater MBH has been associated with reduced post-thermoforming thickness in single-layer aligners (6, 18), its effect on dual-layer aligners remains unclear. The aim of this *in vitro* study was therefore to evaluate the effect of four MBH levels (2, 4, 6, and 8 mm) on the thickness distribution of thermoformed Zendura FLX aligners.

## Methods

### Study design and specimen preparation

This *in vitro* study evaluated the effect of MBH on the thickness distribution of thermoformed Zendura FLX clear aligners. A total of 40 aligners were fabricated and equally allocated into four groups according to MBH: 2, 4, 6, and 8 mm (*n* = 10 per group). The sample size was based on previous *in vitro* studies of thermoformed clear aligners that used a similar number of specimens per group, together with practical considerations related to aligner fabrication and thickness measurements (6, 18). As this was an exploratory laboratory study, no *a priori* power analysis was performed. MBH was defined as the perpendicular vertical distance between the gingival margin of the digital model and the base plane of the printed model. MBH was adjusted digitally before model fabrication using 3Shape Ortho System (version 2023.1, 3Shape A/S, Copenhagen, Denmark), and the digital models were then prepared for three-dimensional printing. Four standardized maxillary digital models corresponding to each MBH were designed using computer-aided design software and fabricated using a Rayshape Edge E2 3D printer (Rayshape, China) with Rayshape Dental Model XE resin. All printed models were cleaned and post-cured in accordance with the manufacturer's instructions. The printed models are illustrated in Figure 1. For each MBH group, ten separate physical models were fabricated from the same digital model, resulting in a total of forty printed models. Each printed model was used for a single thermoforming procedure to avoid potential dimensional changes caused by repeated thermoforming.

**Figure 1:**
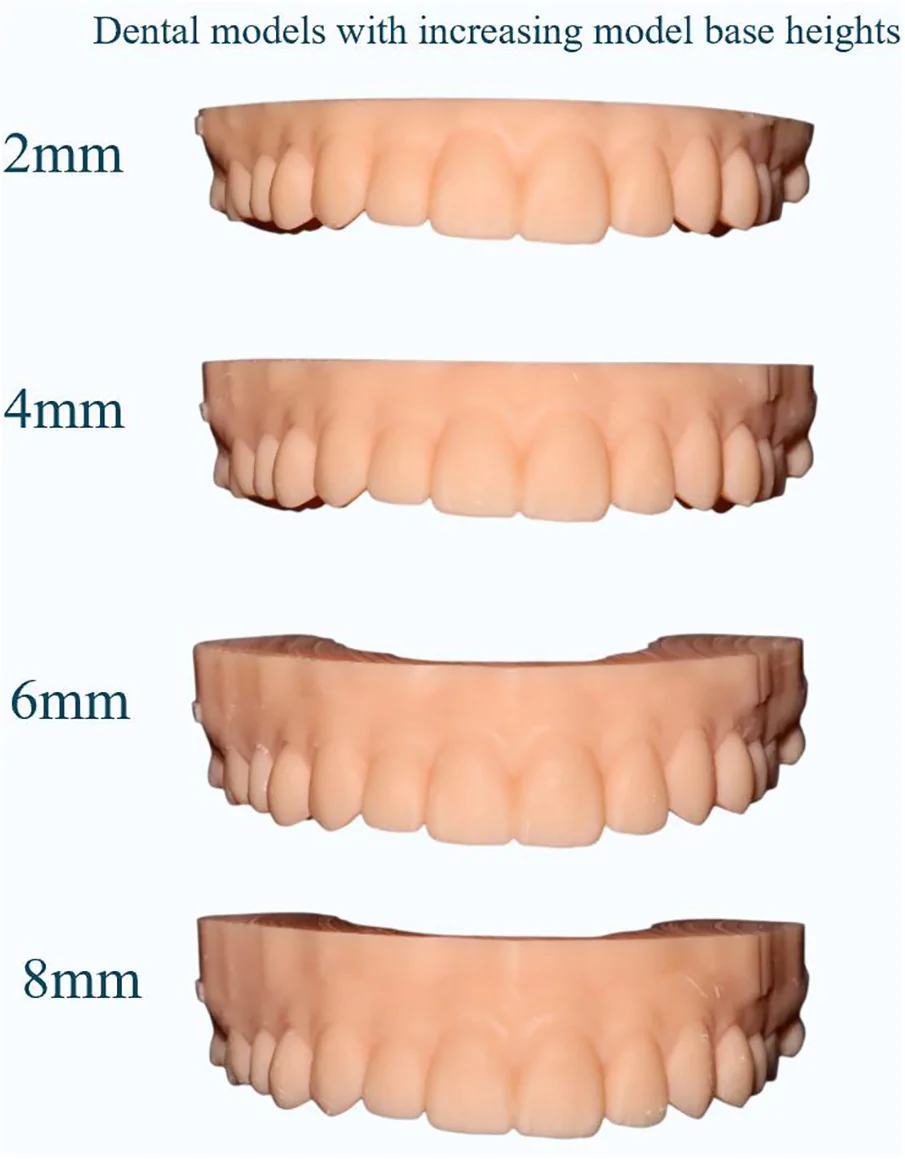
Four dental models with base heights of 2, 4, 6, and 8 mm (top to bottom).

### Aligner fabrication

Each model was centrally positioned on the platform of a pressure-forming unit (Drufomat Scan, Dreve, Germany). Zendura FLX thermoplastic sheets with a nominal thickness of 760 µm were heated at 220 °C for 50 s and thermoformed over the printed models under positive compressed-air pressure (6 bar). The thermoforming parameters, including heating temperature, heating duration, and cooling protocol, were selected according to the manufacturer's recommendations for Zendura FLX material. Following thermoforming, the aligners remained under pressure within the forming unit during a 60 s cooling phase before pressure release and specimen removal.

All fabrication and measurement procedures were conducted in a temperature-controlled laboratory environment maintained at 23 ± 1 °C with approximately 50% relative humidity. Prior to measurement, all thermoformed aligners were allowed to equilibrate at room temperature for at least 30 minutes to minimize potential thermal distortion.

### Thickness measurement protocol

Four reference lines were marked on each aligner using a 0.3 mm fine-tip marker to establish a standardized coordinate system. The buccal longitudinal axis was defined as a vertical line passing through the center of the buccal surface from the incisal edge or cusp tip toward the cervical region, with a corresponding parallel line on the palatal surface defining the palatal longitudinal axis. The buccal transverse axis was drawn horizontally across the greatest mesiodistal width of the buccal surface, connecting the mesial and distal contours, with a corresponding transverse axis on the palatal surface. Measurement sites were identified at the intersections of these reference lines and at relevant anatomical landmarks.

Aligner thickness was assessed at tooth-specific sites across four representative maxillary teeth. For the maxillary central incisor (hereafter called anterior) (tooth 11) and canine (tooth 13), thickness was measured at five sites: incisal edge (I), facial long-axis (FLA), facial-cervical (FC), palatal long-axis (PLA), and palatal-cervical (PC). For the first premolar (tooth 14), thickness was recorded at the buccal (B) and palatal (P) cusp tips in addition to four surface sites (FLA, FC, PLA, and PC). For the first molar (tooth 16), thickness was assessed at the mesiobuccal (MB), distobuccal (DB), mesiopalatal (MP), and distopalatal (DP) cusp tips together with the four surface sites (FLA, FC, PLA, and PC). Cusp-tip measurements were subsequently pooled to derive an occlusal surface (OS) value for posterior teeth: OS for the premolar comprised B and P measurements, while OS for the molar comprised MB, DB, MP, and DP measurements. Detailed definitions of all measurement sites are provided in Table 1. A schematic illustration of all anatomical measurement sites is provided in Figure 2 to facilitate visualization of the measurement protocol.

**Table 1:** Anatomical reference sites used for aligner thickness measurements.

Reference point	Definition	Aggregated location	Assessed teeth
I	Most coronal central point of the incisal edge	Incisal	11, 13
FLA	Long-axis point of the facial surface	Facial	11, 13, 14, 16
FC	Most cervical point of the facial surface	Facial-cervical	11, 13, 14, 16
PLA	Long-axis point of the palatal surface	Palatal	11, 13, 14, 16
PC	Most cervical point of the palatal surface	Palatal-cervical	11, 13, 14, 16
B	Buccal cusp tip	Cusp tip (OS)	14
P	Palatal cusp tip	Cusp tip (OS)	14
MB	Mesiobuccal cusp tip	Cusp tip (OS)	16
DB	Distobuccal cusp tip	Cusp tip (OS)	16
MP	Mesiopalatal cusp tip	Cusp tip (OS)	16
DP	Distopalatal cusp tip	Cusp tip (OS)	16
OS	Pooled cusp-tip measurements	Occlusal surface	14, 16

**Figure 2:**
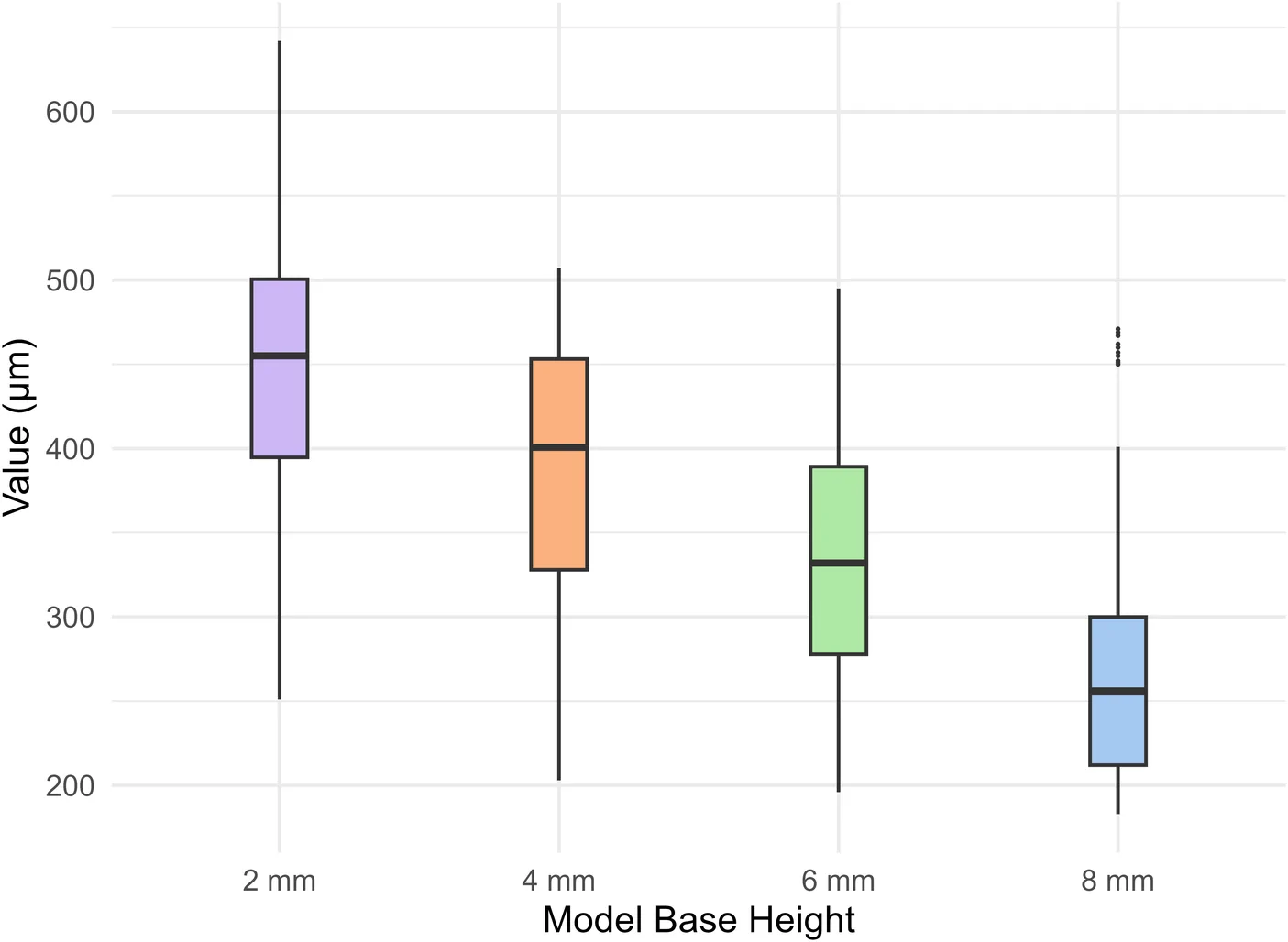
Graphical representation of the measurement sites.

Thickness measurements were obtained using a digital caliper (Panme Syntek, MTM Precision, China; resolution 0.001 mm; range 0–25 mm). Although micro-computed tomography (micro-CT) provides comprehensive three-dimensional thickness mapping, the objective of this study was to compare thickness at predefined anatomical landmarks rather than generate a full thickness map. Therefore, digital caliper measurements were considered appropriate for the study objectives and are consistent with previous thermoforming studies. Micro-CT was not available at our institution during the study period.

All measurements were performed by a single trained operator under academic supervision from the Department of Materials Engineering, Ho Chi Minh City University of Technology. Although thermoformed aligners have a curved geometry, thickness measurements were performed only at predefined anatomical landmarks rather than across the entire aligner surface. This point-based measurement approach has been adopted in previous thermoforming studies for localized thickness assessment (18). The operator underwent calibration training under the supervision of an experienced metrology specialist. To minimize measurement error, the probe was positioned perpendicular to the local aligner surface at each predefined landmark. Duplicate measurements were obtained at every site, and when the difference exceeded 20 μm, a third measurement was performed and the median value was recorded. Intra-operator reliability was assessed by repeated measurements of all 10 aligners (24 sites per aligner) after a 2-week interval and demonstrated good agreement (ICC=0.87).

### Statistical analysis

Data normality was assessed using the Shapiro–Wilk test and quantile-quantile plot, and homogeneity of variances was evaluated using Levene's test. As pooled data across MBH groups, tooth types, and measurement sites demonstrated non-normal distributions, overall thickness values were reported as median and interquartile range (IQR). However, data within each specific combination of MBH, tooth type, and measurement site (*n* = 80 subgroups) were normally distributed in all cases.

For the primary analyses, MBH, tooth type, measurement site, and their interactions were treated as fixed effects. Because multiple measurements were obtained from each aligner, aligner identity was included as a random intercept to account for within-aligner correlation. Alternative random-effects structures, including a nested random intercept for tooth within aligner, were evaluated using Akaike Information Criterion (AIC). As the more complex model did not meaningfully improve model fit, the simpler random-intercept model was retained. When Linear mixed-effects models (LMM) residuals violated normality assumptions, generalized linear mixed-effects models (GLMM) with a gamma distribution and log link function were employed. Model adequacy was assessed by examining model convergence and residual diagnostics. For GLMMs, Pearson residuals, dispersion, heteroscedasticity, and influential outliers were additionally evaluated. Estimated marginal means (EMMs) and 95% confidence intervals (CIs) were reported for all models. *Post-hoc* pairwise comparisons were conducted using Tukey's honestly significant difference (HSD) test to identify specific between-group differences. All statistical analyses were performed with R statistical software (version 4.5.1; R Foundation for Statistical Computing, Vienna, Austria) with the *dplyr, lme4, lmerTest, emmeans*, *DHARMa*, and *ggplot2* packages. Statistical significance was defined as *p* < 0.05.

## Results

### Thickness by model base height

#### Descriptive analysis

The distributional shape of aligner thickness varied across MBH levels (Figure 3). As MBH increased from 2 mm to 8 mm, the central tendency of thickness values decreased, while greater dispersion was observed at lower MBH levels. The distribution exhibited skewness in varying directions depending on the group. Despite these shifts, substantial overlap between groups was observed.

**Figure 3:**
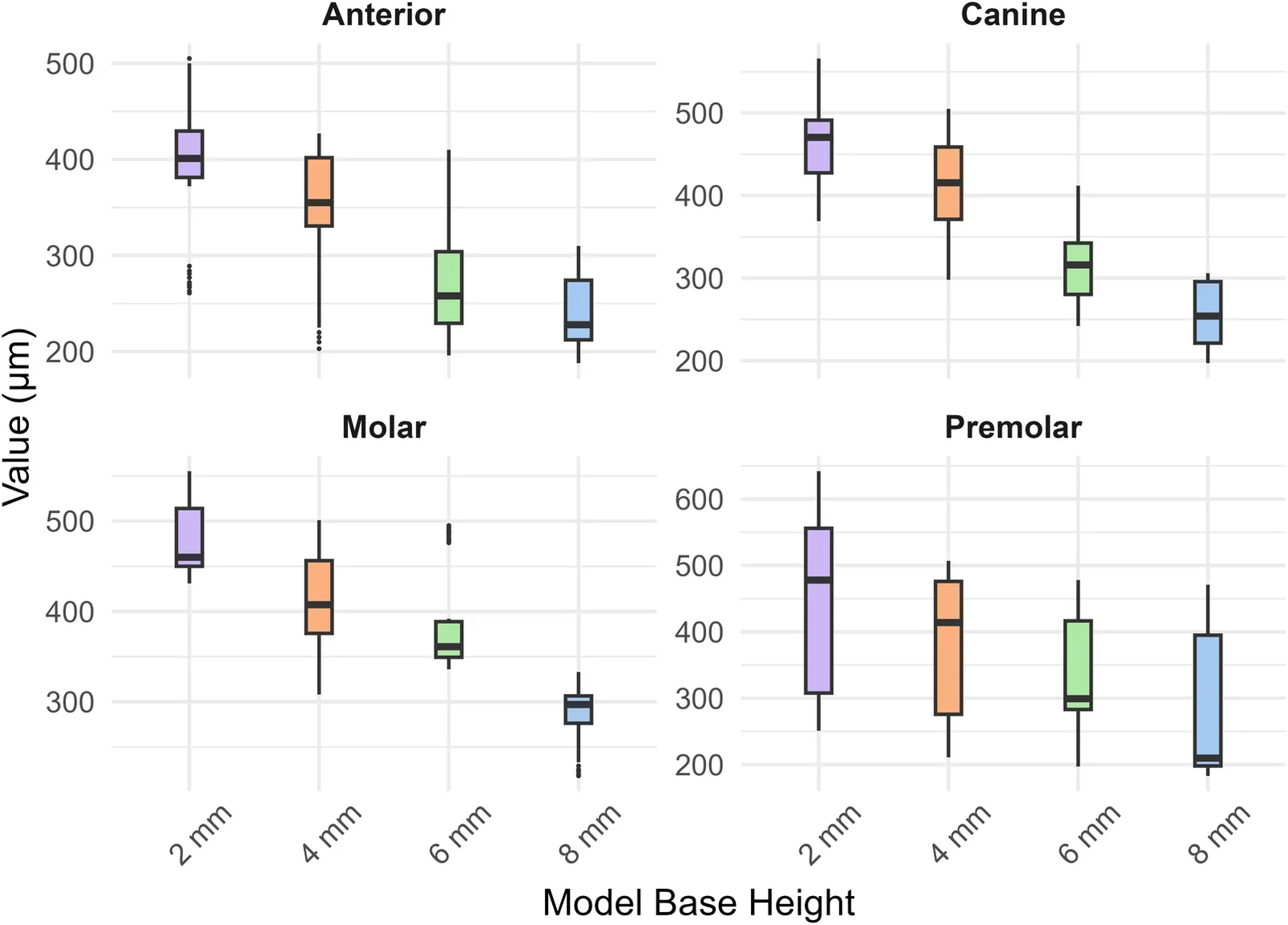
Thickness values by model base height.

#### Comparative analysis

Based on gamma GLMM, EMM thickness decreased gradually with increasing MBH. The EMM thickness was 446 µm (95% CI 432–460) for MBH 2 mm, 386 µm (95% CI 374–399) for MBH 4 mm, 329 µm (95% CI 319–340) for MBH 6 mm, and 268 µm (95% CI 260–277) for MBH 8 mm. Relative to MBH 2 mm, EMM thickness was 15% lower at MBH 4 mm, 35% lower at MBH 6 mm, and 66% lower at MBH 8 mm. Tukey-adjusted pairwise comparisons on log scale indicated that all MBH levels differed significantly from each other (all *p* < 0.001).

### Thickness by model base height and tooth type

#### Descriptive analysis

Across all tooth types, measured thickness values decreased progressively with increasing MBH (Figure 4). The distributions were asymmetric and varied in shape across groups, with particularly wide interquartile ranges observed in premolars. Outliers were noted, particularly in anterior teeth at MBH 2 mm and molars at MBH 6 mm.

**Figure 4:**
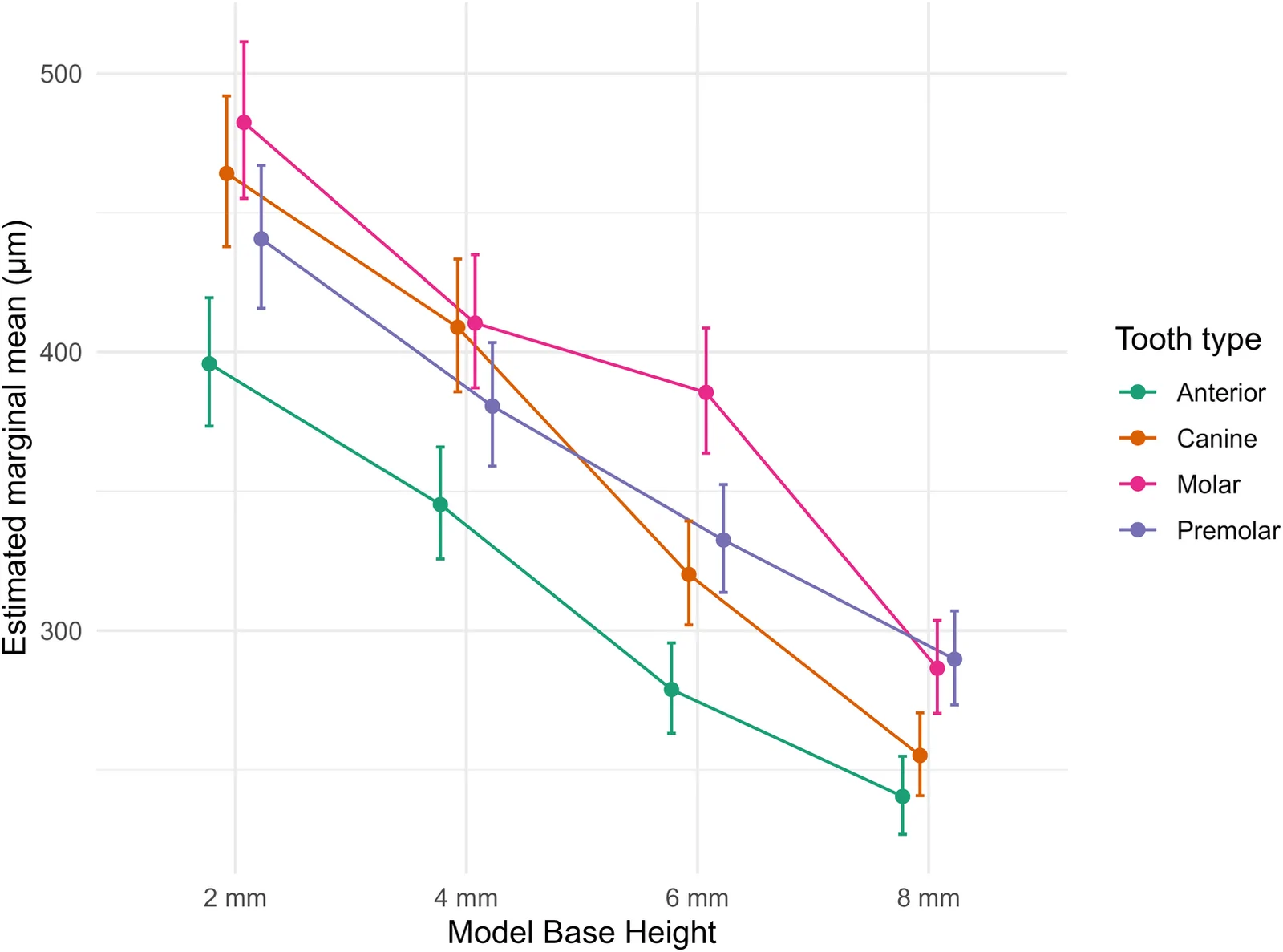
Thickness values of tooth type by model base height.

#### Comparative analysis

EMMs from the Gamma GLMM indicated a consistent decrease in thickness from MBH 2 mm to 8 mm across all tooth types. For anterior teeth, the estimated mean decreased from 396 µm (95% CI 373–420) at MBH 2 mm to 240 µm (95% CI 227–255) at MBH 8 mm. Similar decreasing trends were observed for canines, molars, and premolars (Figure 5).

**Figure 5:**
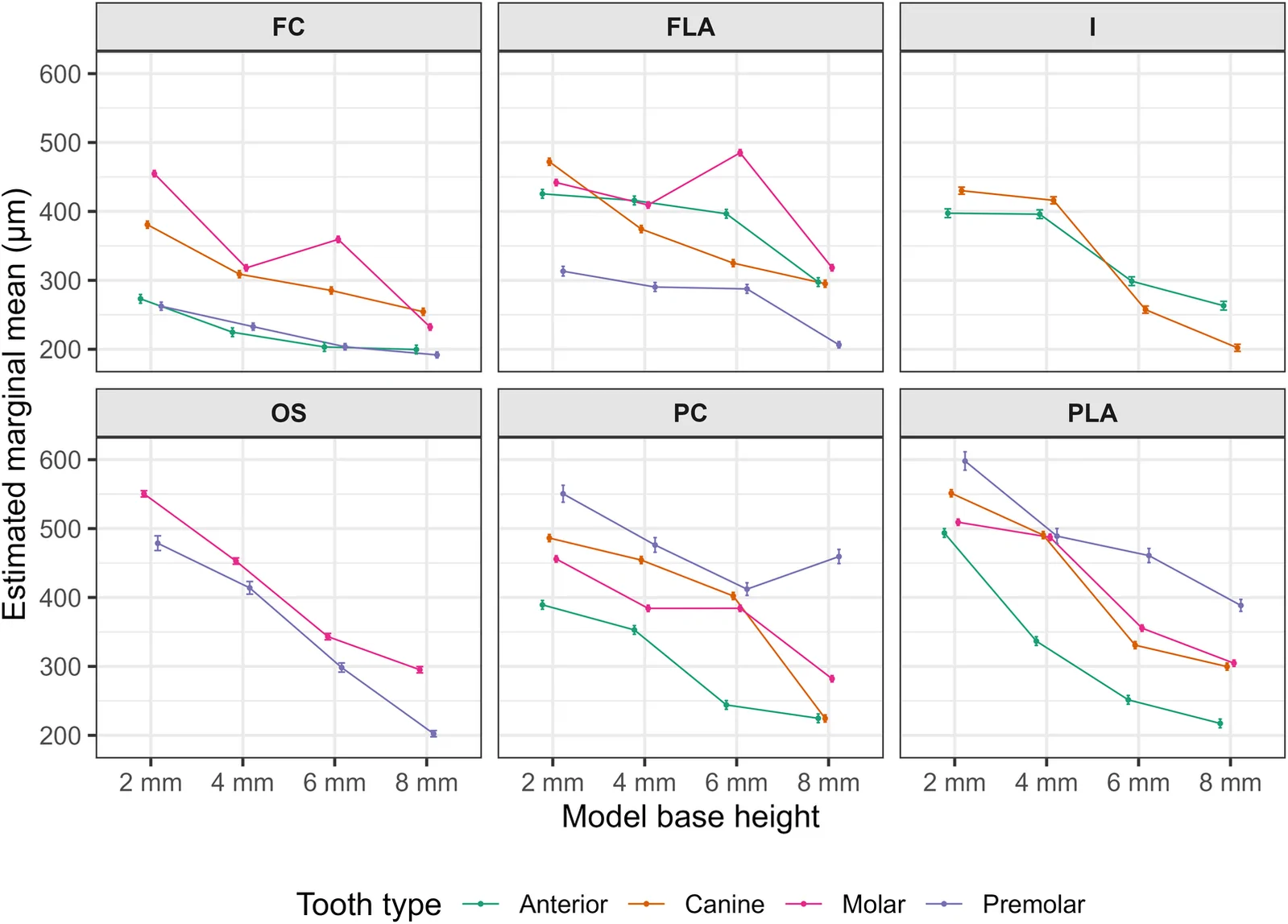
Estimated marginal means from the gamma GLMM of tooth types by model base height.

Tukey-adjusted pairwise comparisons showed that all MBH levels differed significantly from each other for anterior, canine, and premolar teeth (all adjusted *p* < 0.05). For molars, the contrast between 4 mm and 6 mm MBH was not statistically significant (*p* = 0.44), whereas all other contrasts were significant (Supplementary Data Sheet 1).

### Thickness by model base height, tooth type, and measurement site

#### Descriptive analysis

Figure 6 presents boxplots of the raw measurements stratified by MBH, tooth type, and measurement site. Across all tooth types and sites, the distributions showed a systematic decrease in thickness with increasing MBH. Palatal sites (PC and PLA) consistently exhibited higher medians and wider IQR than facial sites (FC and FLA). Right skewness was evident in several groups, particularly at lower MBH levels.

**Figure 6:**
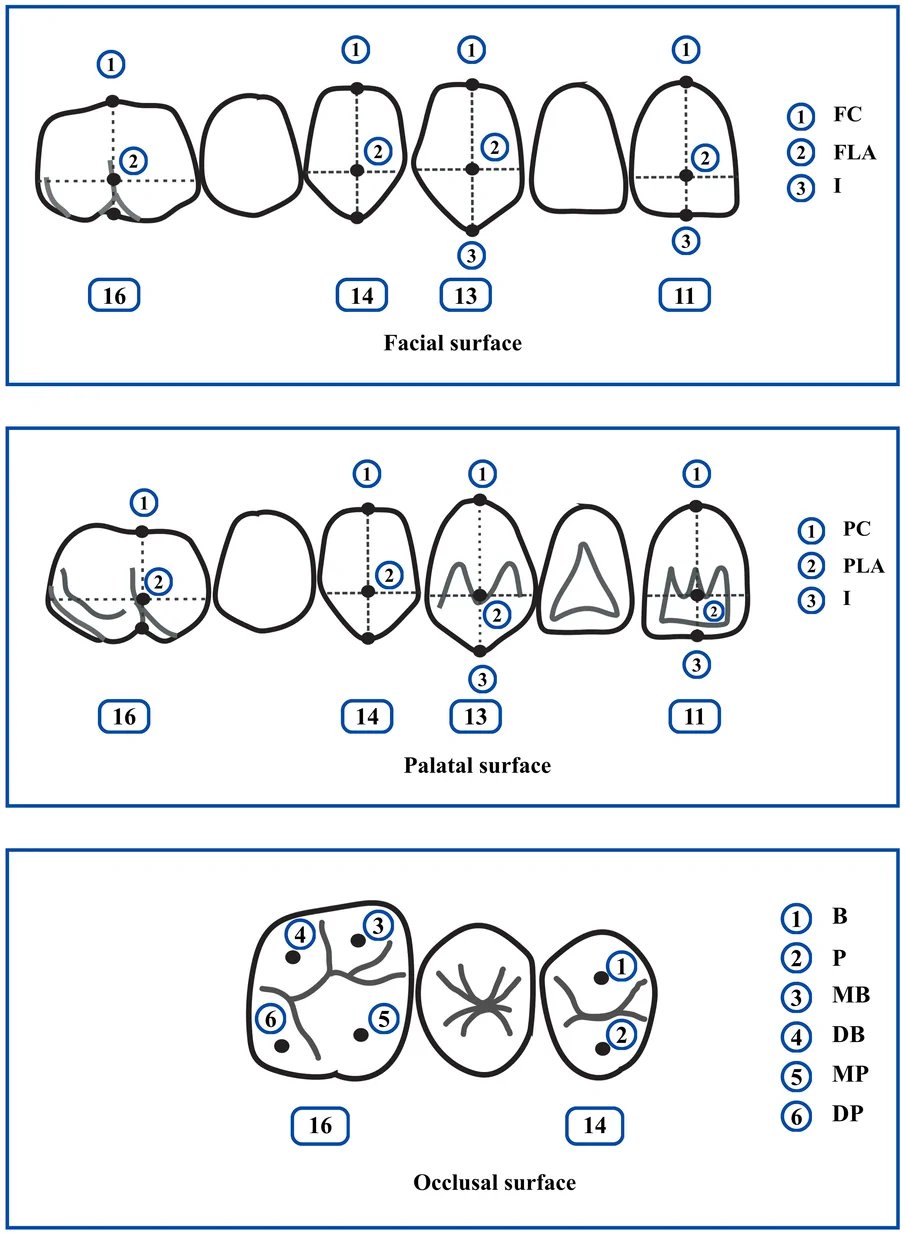
Thickness values at measured sites by model base height, tooth type, and measurement site.

#### Comparative analysis

Across all four tooth types, EMMs decreased progressively with increasing MBH at nearly all sites. For anterior teeth, the highest EMMs were consistently observed at MBH 2 mm across all sites, with particularly elevated values at PLA and PC, while FC showed the lowest values. Similar spatial gradients were observed for canines, premolars, and molars, with posterior sites (PC and PLA) showing larger EMMs than anterior sites (Figure 7 and Supplementary Data Sheet 1).

**Figure 7:**
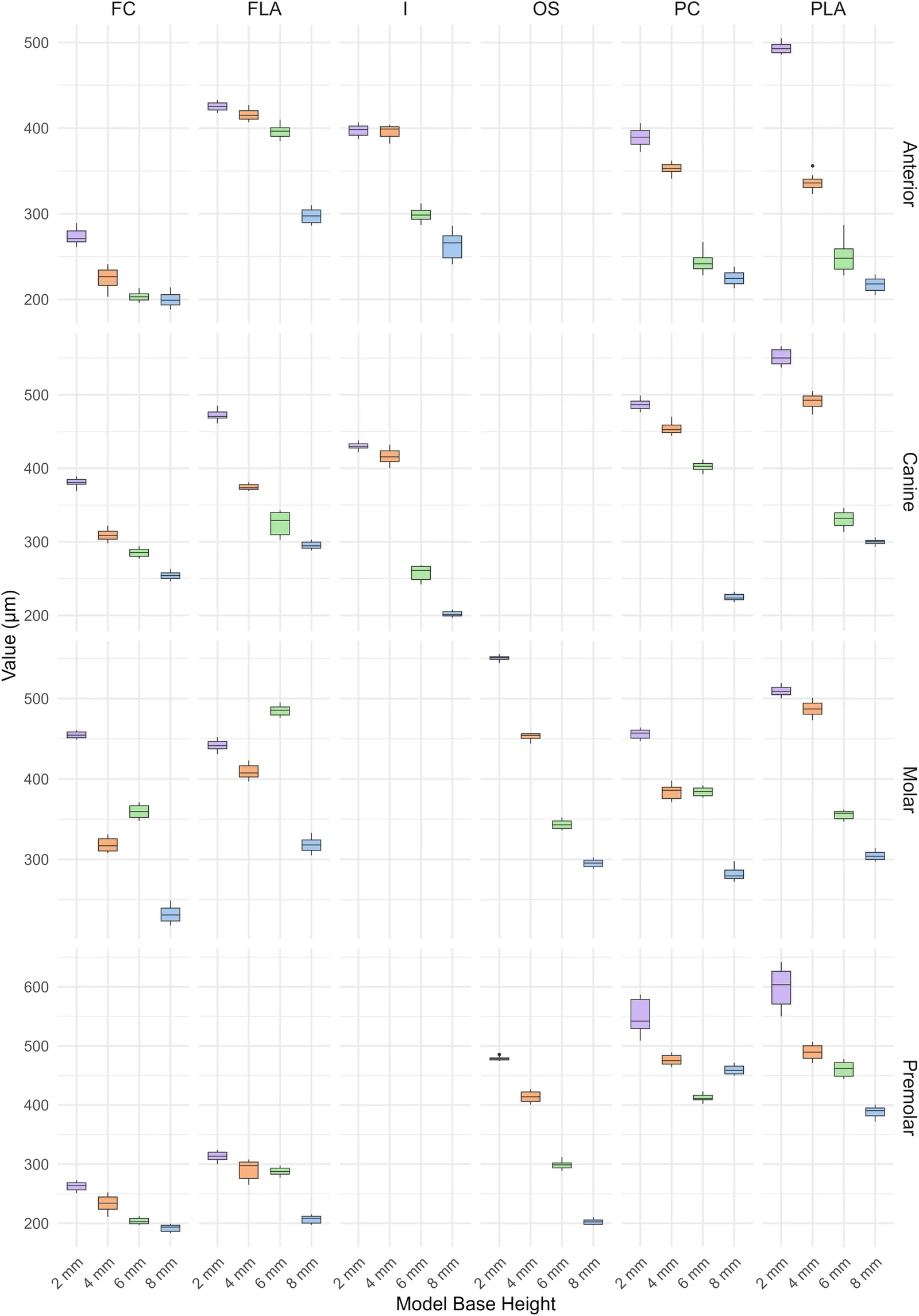
Estimated marginal means of aligner thickness across MBH levels for each tooth type by measurement site. Points represented model-based means and vertical bars indicated 95% confidence intervals derived from the LMM (anterior, canine, molar teeth) and Gamma GLMM for premolar teeth. Distinct trajectories across MBH levels highlight a significant MBH×tooth×site interaction.

For anterior teeth, the LMM model revealed the effect of MBH on thickness differed across measurement sites, with approximately normally distributed residuals (*p* = 0.07). Across all sites, a general decreasing trend in EMMs was observed with increasing MBH, though the magnitude varied by site (Figure 7). All pairwise differences between MBH levels at every site were statistically significant (*p* < 0.001), with the following exceptions: MBH 6 mm vs. 8 mm at the FC site (*p* = 0.85), MBH 2 mm vs. 4 mm at the FLA site (*p* = 0.15), and MBH 2 mm vs. 4 mm at the I site (*p* = 0.99; Supplementary Data Sheet 2).

For canines, the LMM model with normally distributed residuals (*p* = 0.39) showed a progressive decrease in EMMs with increasing MBH (all *p* < 0.001). Pairwise comparisons revealed significant differences between all MBH levels within each site (all *p* ≤ 0.001). The largest MBH-related differences were observed at the I, PC, and PLA sites, e.g., at the I site, the estimated difference between MBH 2 mm and MBH 8 mm was 228.0 µm (*p* < 0.001), while differences at FC and FLA were comparatively smaller (Figure 7, Supplementary Data Sheet 1, and Supplementary Data Sheet 2).

For molars, the LMM model revealed a significant main effect of MBH across measurement sites (all *p* < 0.001) with normally distributed residuals (*p* = 0.41). At all sites, pairwise comparisons between MBH levels were significant (all adjusted *p* < 0.001), except for MBH 4 mm vs. 6 mm at the PC site (*p* = 1.00; Supplementary Data Sheet 2). MBH 2 mm was associated with significantly higher thickness compared with MBH 4, 6, and 8 mm across all sites. The largest contrasts were observed between MBH 2 mm and MBH 8 mm, particularly at OS and PLA sites (Figure 7 and Supplementary Data Sheet 1).

For premolars, EMMs derived from the gamma GLMM indicated a consistent decrease in thickness from MBH 2 mm to 8 mm across all measurement sites. Nearly all pairwise comparisons between MBH levels were statistically significant (*p* < 0.01), with the following exceptions: MBH 4 mm vs. 6 mm at the FLA site (*p* = 0.94) and MBH 4 mm vs. 8 mm at the PC site (*p* = 0.12).

## Discussion

### Inverse association between model base height and aligner thickness

The present study demonstrated a consistent inverse association between model base height (MBH) and the thickness of thermoformed Zendura FLX dual-layer aligners. Both descriptive and mixed-effects analyses revealed a progressive reduction in aligner thickness as MBH increased from 2 mm to 8 mm. These findings are consistent with previous studies reporting thermoforming-related thinning in clear aligners (4, 5). Ryu et al. (5) showed that thermoforming alters the physical characteristics of thermoplastic aligner materials, whereas Bucci et al. (4) reported clinically relevant thickness reduction after thermoforming and intraoral exposure.

The observed reduction in thickness with increasing MBH is also in agreement with previous investigations evaluating model height during thermoforming. Ihssen et al. (6) demonstrated, using micro-CT analysis, that aligners thermoformed on models with greater base height exhibited more pronounced thinning than those formed on models without a base. Similarly, Ghoraba et al. (9) reported progressive thickness reduction in Zendura FLX aligners with increasing model height. Although the inverse relationship between forming depth and aligner thinning has been previously described, existing evidence has mainly focused on single-layer materials, broader model-height ranges, or limited measurement regions. The present study expands upon previous work by evaluating spatial thickness distribution in a dual-layer thermoplastic material across multiple tooth-specific regions and clinically relevant MBH levels.

The reduction in thickness appeared non-linear, with attenuation of thinning beyond 6 mm MBH. This pattern may reflect viscoelastic and thermomechanical behavior during thermoforming, in which material stretching approaches the deformation capacity of the thermoplastic sheet (5). Greater variability was also observed at lower MBH levels, particularly at 2 mm. This finding may be related to less uniform polymer flow during shallow thermoforming conditions (5, 6). Collectively, these observations support the concept that aligner thickness distribution is strongly influenced by geometric and thermoforming-related factors inherent to the fabrication process (4, 6, 7, 9).

### Mechanisms underlying thermoforming-induced thickness variation

Thermoforming-induced thickness reduction is mainly caused by stretching of the thermoplastic sheet during pressure forming (5, 6). As MBH increases, the sheet must stretch over a greater distance before contacting the model surface, resulting in greater material stretching and consequently greater thickness reduction (6, 18).

The behavior of Zendura FLX may also differ from that of conventional single-layer PETG materials because it consists of a rigid PETG outer layer bonded to a more flexible TPU inner layer (19). The PETG layer provides dimensional stability, whereas the TPU layer is more elastic. During thermoforming, the two layers may deform differently but remain bonded together, which may produce a different thickness distribution from that of single-layer PETG aligners. Although layer-specific deformation was not evaluated in this study, these material characteristics may partly explain the observed findings.

Thickness variation may also be influenced by differences in stress distribution during thermoforming (19, 20). Areas with greater curvature and greater forming depth are likely to undergo more stretching and therefore become thinner, whereas areas that contact the model earlier undergo less stretching and retain more thickness. This may explain why palatal regions consistently remained thicker than facial and cervical regions.

### Comparative analysis across model base heights

Comparative analyses indicated that intermediate MBH levels (4–6 mm) were associated with more homogeneous thickness distributions, as reflected by narrower interquartile ranges and fewer outliers. In contrast, the 2-mm MBH group demonstrated greater dispersion and positive skewness, whereas the 8-mm MBH group exhibited pronounced thinning and left-skewed distributions. Similar observations have been reported in previous thermoforming studies, suggesting that both insufficient and excessive forming depths may contribute to uneven material stretching (6, 9).

Although the 6-mm MBH group showed slightly lower variability in several regions, aligners thermoformed on this base height were consistently thinner than those produced on 4-mm models, particularly at palatal and cervical sites. In contrast, the 4-mm MBH maintained comparatively greater thickness while still demonstrating relatively uniform spatial distribution. Within the range evaluated in this study, the 4-mm MBH therefore appeared to provide a reasonable balance between thickness preservation and thickness uniformity. Nevertheless, these findings should be interpreted as laboratory observations rather than direct indicators of clinical performance.

### Thickness variation across tooth types and measurement sites

The present study demonstrated heterogeneous thickness patterns across tooth types and anatomical regions. Posterior teeth generally retained greater thickness than anterior teeth, which is consistent with previous reports suggesting that local surface curvature and thermoforming depth influence the degree of material stretching (7, 12). Premolars exhibited greater variability in thickness values, particularly at lower MBH levels, possibly reflecting differences in crown morphology and localized deformation patterns (7). Canines demonstrated comparatively stable thickness profiles across intermediate MBH levels, whereas molars showed limited differences between MBH 4 mm and 6 mm at selected sites, potentially due to the greater occlusal bulk and complex crown morphology of molars.

Site-specific analyses further demonstrated non-uniform thinning patterns across aligner surfaces. Facial-cervical regions exhibited the greatest thinning, whereas palatal regions consistently retained greater thickness. Similar regional variations have been reported in previous micro-CT and thermoforming studies (7, 9, 12). The significant interaction between MBH, tooth type, and measurement site observed in the present study suggests that thermoforming-induced deformation is spatially heterogeneous rather than uniform throughout the appliance. Certain pairwise comparisons did not reach statistical significance, particularly between adjacent MBH levels at selected anatomical regions. These findings may indicate that local tooth morphology and surface geometry partially modulate the influence of MBH on aligner stretching behavior.

### Clinical and practical implications

The present findings suggest that MBH should be considered an important laboratory parameter during thermoformed aligner fabrication. Previous studies have reported associations between aligner thickness and force transmission characteristics in thermoplastic materials (10, 11). However, because biomechanical force delivery was not directly evaluated in the present study, the observed thickness variations should not be interpreted as direct evidence of altered orthodontic force magnitude or clinical tooth movement.

Although a 2-mm MBH remains commonly used in many manufacturing settings, the present results suggest that shallow bases may be associated with greater thickness variability, possibly due to less uniform material stretching. In contrast, aligners thermoformed on 6-mm and 8-mm models demonstrated greater overall thinning across multiple anatomical regions. Within the range evaluated, the 4-mm MBH appeared to provide a comparatively balanced thickness distribution while avoiding excessive thinning, although the optimal MBH may vary depending on specific laboratory and clinical conditions.

From a manufacturing perspective, incorporating standardized MBH parameters into computer-aided design and thermoforming workflows may help improve fabrication consistency and reduce inter-batch variability across laboratories (9). In addition, emerging direct 3D-printed aligner technologies may allow more controlled regional thickness modification without conventional thermoforming-related deformation (13). Nevertheless, further laboratory and clinical validation studies are required before definitive recommendations can be established.

### Strengths and limitations

The present study evaluated four MBH levels across multiple tooth types and anatomical measurement sites using hierarchical mixed-effects models, providing a detailed characterization of spatial thickness variation in dual-layer thermoformed aligners (4, 6, 9). Several limitations should nevertheless be acknowledged. First, the *in vitro* design could not replicate intraoral conditions such as thermal cycling, saliva exposure, and masticatory loading (4). Second, the findings are specific to Zendura FLX material and may not be generalizable to other thermoplastic systems (5). Third, thickness measurements were obtained using a digital caliper rather than micro-CT imaging, although strict calibration and reliability protocols were implemented (6, 7, 9, 12). Although digital calipers cannot provide continuous three-dimensional thickness mapping comparable to micro-CT, the standardized point-based protocol adopted in this study provided reproducible localized measurements appropriate for comparative evaluation among experimental groups. In addition, force transmission and biomechanical behavior were not directly evaluated. Future studies should incorporate micro-CT analysis, mechanical testing, and evaluate different thermoplastic materials, aligner designs, initial material thicknesses, and thermoforming systems to determine how thermoforming-induced thickness changes influence orthodontic force delivery and clinical tooth movement.

## Conclusion

MBH was identified as an important determinant of thickness distribution in thermoformed Zendura FLX clear aligners. Increasing MBH resulted in a progressive, non-linear reduction in aligner thickness across all tooth types and measurement sites. Among the four levels evaluated, an MBH of approximately 4 mm appeared to provide a comparatively balanced thickness distribution. However, the optimal MBH may vary depending on specific laboratory and clinical conditions. Further studies incorporating intraoral aging, micro-CT assessment, and biomechanical evaluation are warranted.

## Data Availability

The original contributions presented in the study are included in the article/Supplementary Material, further inquiries can be directed to the corresponding author.
